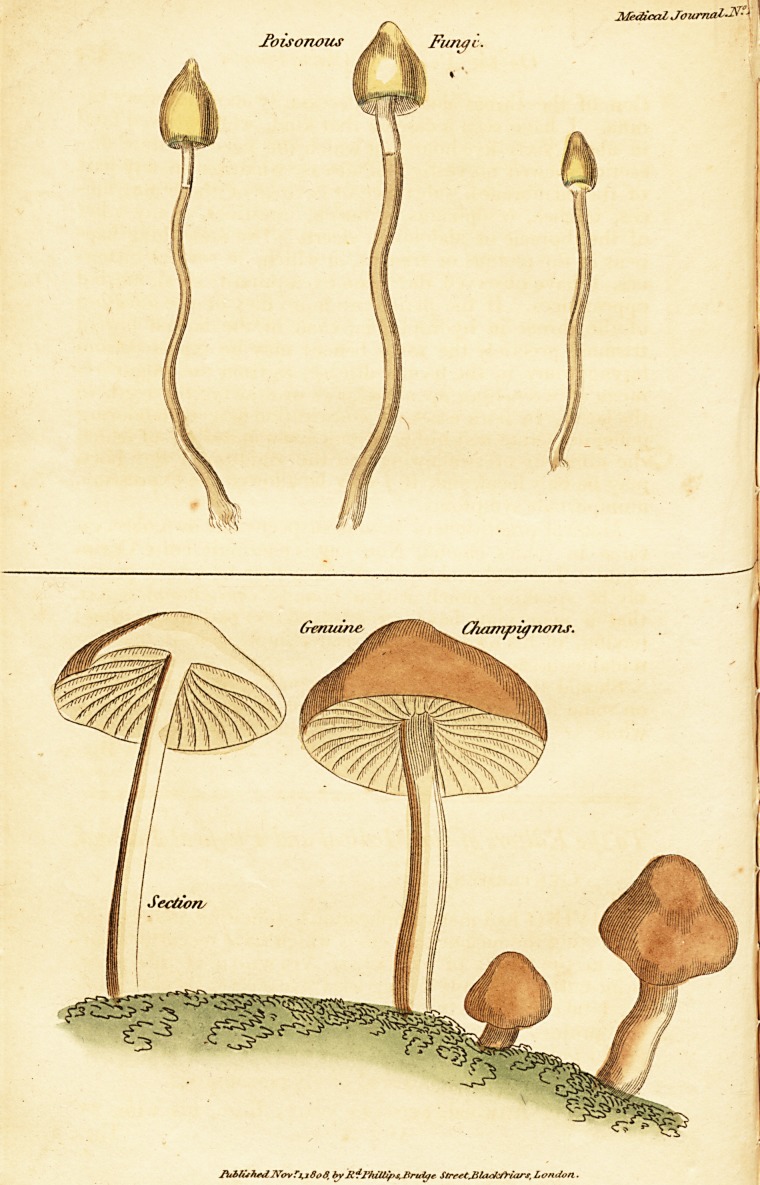# On the Poison of Champignons

**Published:** 1808-11

**Authors:** John Parrott

**Affiliations:** Mitcham


### *To the Editors of the Medical and Phyfical Journal.*

GENTLEMEN,

HAVING had many applications, soliciting information respecting the melancholy cases which have recently occurred in the family of WILLIAM ATTWOOD of this place, print-cutter, and not having leisure to answer them severally, I take the liberty of communicating the particulars to the profession in general, through the medium of your useful publication.

The family consisted of six persons, viz.

William Attwood, aged 45 years; Eliza, his wife, 38.

 Their

Their daughters, viz. Mary, 14; Hannah, 11; Sarah, 7; Eliza, 5.

On Monday the 10th instant, they all ate stewed champignons, at one o'clock, which stew was made in an iron vessel, and consisted of the articles already specified, with the addition of butter and flour, pepper, salt, and water only; and each of the parties, (Hannah excepted) was supposed to have eaten more than half a pint. Within ten minutes after they had eaten their meal, they felt their spirits exhilarated, and the eldest daughter said to her mother, "How funny you look." All the parties continued cheerful till about six o'clock, when having taken their tea, they were attacked with stupor, which was not of long continuance; this was soon succeeded by severe pain in the bowels, accompanied with violent vomiting and copious purging, which continued till the following afternoon, when the parents were alarmed, and requested my attendance.

Under these symptoms, it appeared that the first step necessary to be taken, was to get rid of the poison; for which purpose, oily opening medicines were administered, with the use of emollient glysters, and plentiful dilution with warm broths was recommended.

This method of treatment appeared to promise success in the case of Mary, who had so far recovered on the following day, (Wednesday) that she walked into the village about a quarter of a mile from home; in the evening, however, the symptoms returned; on Thursday evening she became convulsed, and died on Friday morning, at two o'clock.

Hannah only ate two spoonfuls of the stew, as she did not like its flavour; this girl recovered after a severe vomiting and purging.

Eliza did not complain much of her sufferings, but became convulsed at the same time her sister Mary did, and died half an hour after her.

Sarah never complained of pain in the head, but was continually suffering under extreme pain in the bowels, which was increased upon pressure, but no tension existed; glysters afforded her no relief, and she died on Saturday morning, in the same convulsed state as her sisters.

Permission having been obtained to open one of the bodies, that of Sarah was examined, as she had suffered under the most excruciating pain in the bowels, but no appearance of disease was manifest in any of the abdominal viscera; the stomach was empty, and also the whole of the alimentary canal.

On Friday the 14th, the vomiting still continuing in the father father and mother, it was thought proper to quiet the irritation of the stomach. For this purpose, small doses of laudanum were administered, but without effect; the effervescing draught was then given, which succeeded, but the pain in the bowels was thereby so much increased, that they both regretted having taken it.

On the same night, Mrs. Attwood miscarried; she was two months advanced in pregnancy; but with her husband, is now in a state of convalescence.

During the progress of this unfortunate occurrence, the pulse in each of the patients was quickened and varied from 100 to 120 strokes in a minute, but it was not sufficiently full to justify the use of the lancet; the tongue was parched and slightly streaked with white; the tunicæ conjunctivæ were not inflamed, and the parties were all perfectly sensible; the urine was secreted in very small quantity, but it was not high coloured.

A dog which had partaken of the stew, died on Wednesday night, apparently in great agonies.

Such, Gentlemen, are the leading circumstances which I have witnessed of the fatal effects of persons having eaten of a particular species of fungus, which they supposed was of the wholesome kind. Were I to attempt to reason thereon, such reasoning, after what I have advanced in the case of Sarah, would prove of the negative kind.

Mr. Wheeler, of St. Bartholomew's Hospital, has procured some of the plants herein described, which he is now examining, and from the extensive Botanical knowledge which this Gentleman is known to possess, the public will probably derive more information upon this subject, than can possibly be expected from the pen of

Your very humble servant,

JOHN PARROTT.

*Mitcham, Oct. 22,* 1808.

---

To Mr. Wheeler's kindness we owe the following information. As soon as he was informed of the event, he repaired to the spot where the mushrooms were gathered, and immediately recognized that variety of the Agaricus, which, on a former occasion, nearly proved fatal.* That he might be certain as to the identity of the fungus, he brought another with him to the distressed parents, who assured

---

* See our Journal, Vol. iii. p. 41.

assured him that it was not that which formed their stew. He then shewed them that which we have marked as poisonous. This, the father instantly declared to be like those of which they had eaten.

The following is the description taken from Sowerby's Nineteenth Number of English Funguses.

"AGARICUS SEMIGLOBATUS. *With.* 3. *ed. v.* 4. 270.
———— GLUTINOSUS. *Curt. Lond. fasc.* 3. *t.* 69.

Common almost every where. It is most generally of a hemisphærical form, yet, like other fungi, occasionally varies, and perhaps may furnish a new argument, that those gathered in wet places or bad weather are unwholesome. The varieties 1, 2, and 3, with the pileus acuminated, are most certainly of this description, and nearly proved fatal to a poor family in Piccadilly, London, who were so indiscreet as to stew a quantity (found in St. James's Green Park) for breakfast. See Mr. Everard Brande's account in the Medical and Physical Journal."

We have added a figure of the *Agaricus Pratensis,* commonly called Champignons in England. These are always found growing in a somewhat circular direction, as if tending to what are called fairy rings.

The poisonous species affects something of the same form, but much more imperfectly. But the most striking peculiarity is the *accuminated pileus,* or the sharpness of the summits.

Mr. Wheeler gathered another fungus on the same spot, which he suspects made a part of the ill-fated repast. It rises about an inch and a half from the ground, at right angles. He has not been able as yet to find it described in any work to which he has referred, but promises us every information he can collect on the subject.

## Figures and Tables

**Figure f1:**